# Challenges and limitations of evaluating the efficacy of music intervention for preterm infants: auditory development, methodological heterogeneity, medical complexity, parental involvement and environmental barriers

**DOI:** 10.3389/fped.2026.1716416

**Published:** 2026-02-26

**Authors:** Vito Giordano, Ji Sun Kim, Shmuel Arnon, Angelika Berger, Christian Gold

**Affiliations:** 1 Division of Neonatology, Pediatric Intensive Care and Neuropediatrics, Department of Pediatrics and Adolescent Medicine, Comprehensive Center for Pediatrics (CCP), Medical University of Vienna, Vienna, Austria; 2 Laniado Hospital Netanya, The Adelson School of Medicine, Ariel University, Ariel, Israel; 3 GAMUT—The Grieg Academy Music Therapy Research Centre, NORCE Norwegian Research Centre AS, Bergen, Norway; 4 Department of Clinical and Health Psychology, University of Vienna, Vienna, Austria

**Keywords:** acoustic, music, neurodevelopment, NICU (neonatal intensive care unit), preterm

## Abstract

Despite growing interest in music therapy (MT) as a supportive intervention in neonatal intensive care units (NICUs), strong evidence for its long-term efficacy remains scarce. This perspective article explores the multifaceted challenges of implementing and evaluating MT in NICUs, particularly for preterm infants. These challenges include (1) limited understanding of premature auditory development, (2) environmental acoustics, (3) methodological inconsistencies in MT delivery, and the complex medical (4) and psychosocial (5) context of the NICU. Further compounding this issue is the underappreciation of parental involvement and the perception of MT among healthcare professionals. Addressing these gaps is essential for establishing standardized, effective MT protocols tailored to this vulnerable population.

## 1 Introduction

Music therapy (MT) in the neonatal intensive care unit (NICU) is a field filled with promise—but also with profound complexity. While studies increasingly highlight short-term benefits of MT for preterm infants, e.g., improved physiological stability (1, 2), evidence of long-term efficacy remains limited (3). This gap is not merely the result of insufficient data collection; it reflects deeper methodological, developmental, and contextual challenges that must be acknowledged if the field is to evolve meaningfully.

### 1.1 Acoustic differences between the womb and the NICU

We still know too little about how preterm infants process sound (4–9). In utero, the fetus hears through fluid—protected from harsh frequencies and surrounded by rhythmic, low-frequency vibroacoustic stimuli such as the maternal heartbeat and voice (10). In contrast, the NICU exposes infants to air-transmitted, high-frequency, often unpredictable sounds.

Moreover, much of what we know about auditory development is based on intrauterine behavioral studies or on term infants and adults (11, 12). The anatomy and resonance of the auditory canal in preterm infants are fundamentally different (8, 11), yet this is rarely considered in either clinical protocols or research design. Even neurophysiological studies measuring auditory evoked potentials report highly variable results, especially before 32 weeks of gestation, when responses are still immature and inconsistently detectable (13). This raises a critical question: what do preterm infants actually perceive?

### 1.2 Acoustic and environmental challenges in the NICU

The NICU presents a uniquely hostile acoustic environment (4, 7, 10); mechanical ventilation, alarms, and equipment noise dominate (10). Incubators, while designed to support life, often distort or block external sounds, including beneficial stimuli like maternal speech or therapeutic music, while at the same time amplifying noises from devices such as ventilators (8, 14). Several studies have noted that, even when incubator doors are open, sound perception inside remains severely limited, especially for infants receiving respiratory support (4, 8, 9, 14). This calls into question the efficacy of standard MT protocols, particularly those involving soft vocalizations or instruments that may not reach the infant effectively or meaningfully.

### 1.3 Methodological heterogeneity: an unsolved barrier

**Figure 1:**
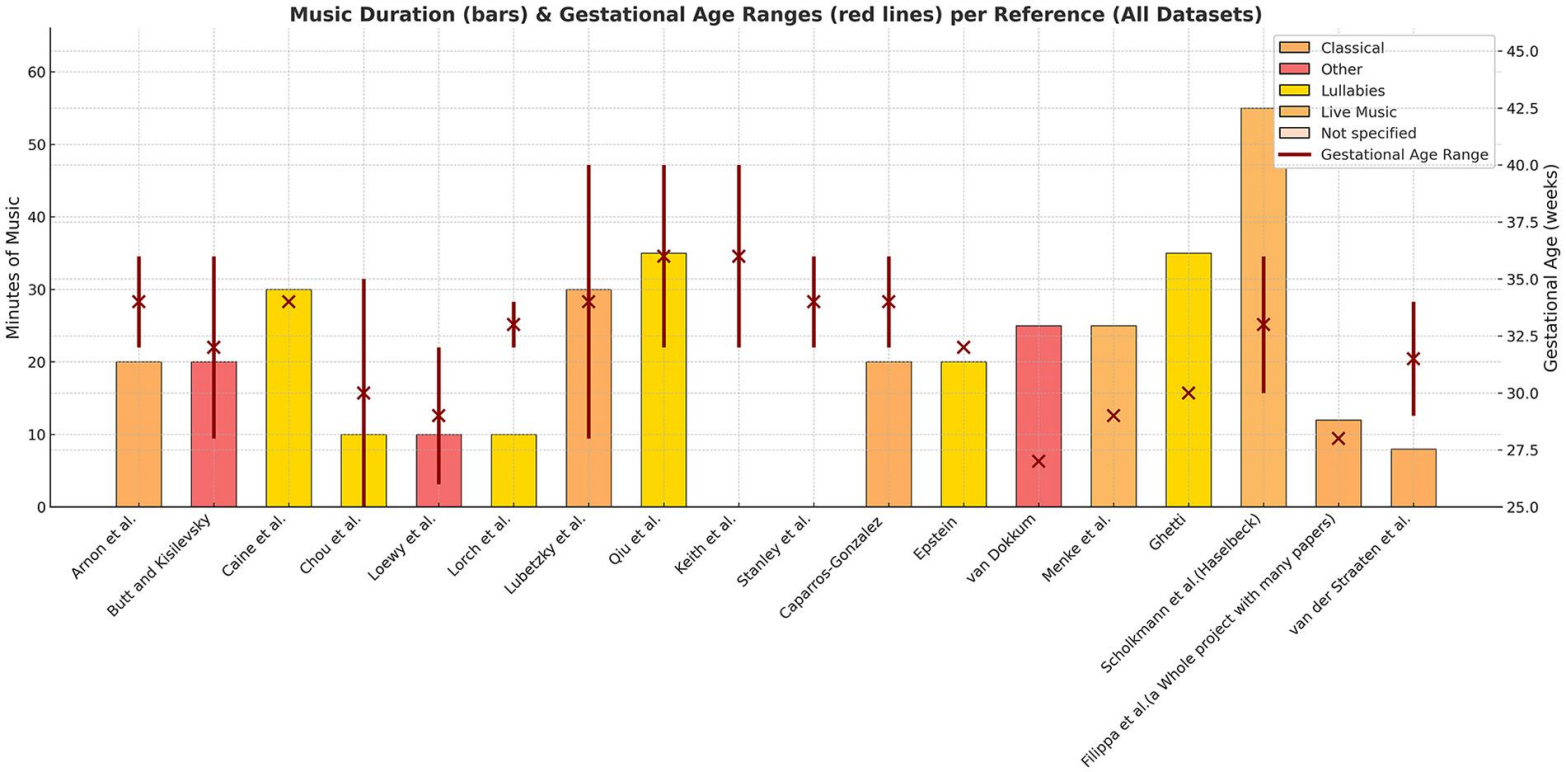
Clinical heterogeneity in studies on music-based intervention outcomes in the NICU (18–31). Studies were selected from an earlier systematic review (16), supplemented with updated searches.

The current MT studies in the NICU vary dramatically in design, making comparisons and generalizations difficult (15–17). Variations in gestational age, post-menstrual age at recruitment, session frequency and duration, sample sizes, and the type of musical intervention—whether delivered by trained music therapists or as pre-recorded music—create considerable heterogeneity and prevent firm conclusions about the sustained benefits of MT (15, 18–31). Figure 1 therefore, illustrates the heterogeneity of music-based intervention studies based on an updated pre-selection derived from Anderson and Patel review (2018). Since the aim of this Perspective manuscript is not to provide a systematic selection of the literature, we relied mostly on their work for the generation of this figure. The Authors, in fact, examined the potential impact of music therapy on the neurodevelopment of preterm infants and identified ten studies out of 140 that met their methodological rigor criteria.

It is crucial to differentiate between music interventions (e.g., playing recorded lullabies in the incubator or bedside) and music therapy conducted by certified music therapists. The latter typically involves individualized assessment, guided parental involvement, real-time improvisation adapted to the infant's physiological cues, and integration into the broader interdisciplinary care team. Such therapeutic frameworks entail a fundamentally different mechanism of action and clinical impact compared to passive listening to pre-recorded music. Failure to explicitly distinguish between these categories in research designs risks conflating their effects and contributes to inconsistent outcomes across studies.

Furthermore, instruments and vocal strategies differ widely, as do therapist training levels and therapeutic goals (32). In many cases, studies fail to report contextual variables such as the infant's respiratory support level, incubator type, or ambient noise during the session—all of which directly influence both delivery and perception of music (15–17).

### 1.4 Medical complexity and developmental variability of patients

The neonatal intensive care unit environment is not static, and neither are its patients. Preterm infants—especially those born at extremely low gestational ages—experience a dynamic and often fragile clinical course. In the earliest stages, the infant's physiological priorities are centered on survival: maintaining respiration, thermoregulation, and hemodynamic stability. As infants progress beyond acute instability, their needs shift toward nutrition, sensory regulation, and interaction with the external environment. This evolving developmental context demands a parallel flexibility in therapeutic interventions, not only in terms of frequency and duration, but also in the type of sensory intervention, as growing babies have different needs compared to early postmenstrual ages (33). Yet most research and clinical practice relies on specific frameworks—often prescribing session of 15 to 20 min per day when possible (34), since not every hospital can afford the regular presence of a music therapist.

Such temporal uniformity is difficult to reconcile with the lived experience of infants in the NICU. A newborn who undergoes 12 to 15 painful or invasive procedures each day (particularly within the first three weeks of life) is subjected to a high level of stress and sensory disruption (35–38). While brief MT sessions may offer immediate physiological benefits—such as stabilization of heart rate or improved oxygen saturation—these effects can be short-lived in the face of repeated noxious stimuli. A single intervention may be insufficient to meaningfully counterbalance the overall sensory burden of the NICU environment. This raises important questions about “therapeutic dosage”: not only how long an individual session should last, but also how often sessions should occur, and at what developmental or clinical stage they are most effective.

Moreover, the infant's stage of development significantly shapes how they receive and respond to MT. Music therapy has also been applied in different populations of interest, but only rarely in extremely preterm.

In the early weeks, especially below 30 weeks' gestational age, sensory systems are still immature. Auditory input may be inconsistently processed or even stressful if not appropriately modulated. As the infant matures, however, opportunities for engagement expand. Music therapy may begin to support more than regulation alone—it can facilitate emerging communication patterns, promote attentional focus, and scaffold caregiver interaction. Yet this gradual transformation—from a critically ill neonate to a socially responsive infant—is not well enough captured in the existing literature, which tends to treat preterm infants as a homogenous group rather than as individuals on distinct developmental trajectories.

Only after considering these developmental layers should attention turn to comorbidities—whose importance cannot be overstated. Infants in the NICU often develop complications such as bronchopulmonary dysplasia, intraventricular hemorrhage, necrotizing enterocolitis, or retinopathy of prematurity, each of which can influence not only medical stability but also sensory tolerance and neurodevelopment. These conditions may necessitate adjustments to MT protocols in terms of volume, frequency, and the type of auditory stimuli used. Importantly, they can also impact the infant's long-term outcomes—the very outcomes that MT seeks to improve.

### 1.5 Neglected dimensions: parental involvement and psychological state

Despite the well-established importance of parent-infant bonding for long-term developmental outcomes, many studies on music therapy in the NICU continue to underrepresent the role and psychological state of parents. Maternal and paternal presence can significantly influence an infant's recovery trajectory, both during hospitalization and post-discharge (23, 39). Their absence—often due to emotional distress, logistical constraints, or insufficient institutional support—can diminish the potential benefits of even the most carefully structured interventions (40).

Adding to this complexity is the ongoing debate over the optimal mode of music delivery: live vs. recorded (15–17). While live music therapy offers real-time responsiveness and the possibility of interpersonal co-regulation, it requires the physical presence of trained professionals—a resource not always available. Recorded music, by contrast, is more easily scalable but raises concerns about sound fidelity, frequency range, and hygienic safety. Many commercially available speakers are not designed for medical environments and may not reproduce the low-frequency components essential for fetal-like auditory experiences. Moreover, concerns about cleaning and infection control can further limit their appropriateness in neonatal settings.

In this context, the potential of parental involvement becomes even more relevant. The use of recorded parental voices or singing has emerged as a feasible and emotionally meaningful alternative, especially when facilitated within a therapeutic framework (15). Beyond sound alone, the inclusion of parents introduces a multi-sensory dimension—combining voice, presence, and touch—that may enhance co-regulation and bonding (23, 41). This human element, often overlooked in strictly auditory-focused studies, is critical in shaping the infant's early sensory and relational world.

Recognizing and integrating these psychosocial and practical dimensions is essential if music therapy is to evolve from an experimental adjunct into a truly developmental and family-centered standard of care.

## 2 Structural and cultural barriers to the integration of music therapy in the NICU

The limited implementation of music therapy (MT) in neonatal intensive care units is not merely a matter of clinical oversight, but rather the result of complex and interwoven structural, cultural, and epistemological barriers (22). Resource constraints—both in terms of staffing and dedicated funding—remain a primary obstacle, particularly in healthcare systems where MT is not yet embedded within standard care protocols. Compounding this challenge is the scarcity of robust longitudinal evidence demonstrating clear long-term benefits, which can make it difficult for administrators and clinicians to justify its systematic inclusion (3).

Beyond logistical constraints, the integration of MT is also influenced by epistemological differences between biomedical and psychosocial intervention models. NICU care traditionally prioritizes interventions with clearly measurable, short-term physiological or neurodevelopmental endpoints, whereas MT effects may be indirect, relational, and developmentally mediated, making them less immediately visible within conventional clinical metrics (15, 16).

Culturally, MT may not align with the predominant biomedical model, which often prioritizes pharmacological and procedural interventions. As a result, some non-pharmacological approaches may be perceived as ancillary or lacking rigor, particularly in high-acuity environments where clinical priorities are narrowly focused on survival and stabilization. Additionally, responses to music are inherently individualized, and this variability can generate uncertainty about efficacy and reproducibility across patient populations.

This perception is further reinforced by the presence of mixed finding in the literature regarding long-term neurodevelopmental outcomes. Systematic reviews analyses have consistently highlighted substantial heterogeneity and low certainty of evidence for sustained developmental benefits, despite more consistent short-term effects on physiological stability or parental outcomes (15–17). Similarly, large randomized trials have demonstrated improvements in parent–infant bonding and stress regulation without corresponding effects on standardized neurodevelopmental scores at later follow-up (3, 23).

Institutionally, NICUs are governed by strict environmental regulations aimed at minimizing noise to preserve sleep quality (42) and ensure clinical concentration. While these priorities are valid, they can inadvertently conflict with the introduction of therapeutic sound-based.

These null or equivocal findings do not necessarily indicate a lack of therapeutic potential, but rather underscore limitations in current outcome frameworks. Standardized neurodevelopmental assessments may be insufficiently sensitive to capture changes in early co-regulation, sensory integration, or relational development—domains in which MT is most plausibly active (16, 41). As a result, interventions with developmentally meaningful but less quantifiable effects risk being undervalued within resource-constrained clinical systems (22).

Furthermore, the lack of standardized protocols specifying how, when, and by whom MT should be delivered limits its integration into daily care routines. Despite these challenges, the growing body of research and the increasing recognition of developmental and family-centered care offer a promising framework for the future. With targeted investment, interdisciplinary collaboration, and greater awareness of the mechanistic underpinnings of MT, it is possible to envision a NICU model in which music is not an exception, but an integrated component of individualized, evidence-informed care.

## 3 Toward a constructive future: recommendations and reflections

The way forward lies in rethinking how we study and deliver MT. Several key priorities emerge:

Refine our understanding of preterm auditory development.
  Investment in basic research on extrauterine auditory processing—considering medium (air vs. fluid), ear canal anatomy, and neurodevelopmental maturity—is essential.
Expand standards for MT practice in the NICU.
  Guidelines must define not only duration and frequency but also acoustic characteristics, therapist qualifications, and patient readiness criteria. For example, patient readiness could include observable signs such as stable vital signs, minimal stress cues (e.g., absence of grimacing or finger splaying), and the ability to tolerate brief periods of stimulation without physiological instability.
Include and support families as active therapeutic agents.
  Parent presence, voice, touch, and emotional wellbeing should be central to MT protocols and outcome assessments.
Improve acoustic conditions in the NICU.
  Technological innovations in incubator design and environmental noise control could significantly enhance the efficacy of sound-based interventions.
Foster interdisciplinary collaboration.
  Integration of MT into standard NICU care requires collaboration, mutual respect, and training across professional boundaries—including medical staff, nurses, psychologists, and therapists.
Design research for the long term.
  Longitudinal studies, though challenging, are essential to understanding the developmental trajectories influenced by early MT exposure. They must also account for comorbidities and environmental variables across time.

## Data Availability

The original contributions presented in the study are included in the article/Supplementary Material, further inquiries can be directed to the corresponding author.
